# No evidence for an effect of working from home on neck pain and neck disability among Swiss office workers: Short-term impact of COVID-19

**DOI:** 10.1007/s00586-021-06829-w

**Published:** 2021-04-04

**Authors:** Andrea M. Aegerter, Manja Deforth, Venerina Johnston, Gisela Sjøgaard, Thomas Volken, Hannu Luomajoki, Julia Dratva, Holger Dressel, Oliver Distler, Achim Elfering, Markus Melloh, the NEXpro collaboration group, Marco Barbero, Beatrice Brunner, Jon Cornwall, Yara Da Cruz Pereira, Tobias Egli, Markus J. Ernst, Irene Etzer-Hofer, Deborah Falla, Michelle Gisler, Michelle Haas, Venerina Johnston, Sandro Klaus, Gina M. Kobelt, Kerstin Lüdtke, Corinne Nicoletti, Seraina Niggli, Salome Richard, Nadine Sax, Katja Schülke, Lukas Staub, Thomas Zweig

**Affiliations:** 1 School of Health Professions, Institute of Health Sciences, Zurich University of Applied Sciences, Winterthur, Switzerland; 2 School of Health and Rehabilitation Sciences, The University of Queensland, Brisbane, QLD Australia; 3 Department of Sports Science and Clinical Biomechanics, University of Southern Denmark, Odense, Denmark; 4 School of Health Professions, Institute of Physiotherapy, Zurich University of Applied Sciences, Winterthur, Switzerland; 5 Faculty of Medicine, University of Basel, Basel, Switzerland; 6 Division of Occupational and Environmental Medicine, Epidemiology, Biostatistics and Prevention Institute, University Hospital Zurich, University of Zurich, Zurich, Switzerland; 7 Department of Rheumatology, University Hospital Zurich, University of Zurich, Zurich, Switzerland; 8 Institute of Psychology, University of Bern, Bern, Switzerland; 9 School of Medicine, The University of Western Australia, Perth, WA Australia; 10 Curtin Medical School, Curtin University, Bentley, WA Australia

**Keywords:** Neck pain, Neck disability, COVID-19, Pandemic, Working from home

## Abstract

**Purpose:**

The aim of this study was to investigate the effect of working from home on neck pain (NP) among office workers during the COVID-19 pandemic.

**Methods:**

Participants from two Swiss organisations, aged 18–65 years and working from home during the lockdown (*n* = 69) were included. Baseline data collected in January 2020 before the lockdown (office work) were compared with follow-up data in April 2020 during lockdown (working from home). The primary outcome of NP was assessed with a measure of intensity and disability. Secondary outcomes were quality of workstation ergonomics, number of work breaks, and time spent working at the computer. Two linear mixed effects models were fitted to the data to estimate the change in NP.

**Results:**

No clinically relevant change in the average NP intensity and neck disability was found between measurement time points. Each working hour at the computer increased NP intensity by 0.36 points (95% CI: 0.09 to 0.62) indicating strong evidence. No such effect was found for neck disability. Each work break taken reduced neck disability by 2.30 points (95% CI:  − 4.18 to  − 0.42, evidence). No such effect was found for NP intensity. There is very strong evidence that workstation ergonomics was poorer at home.

**Conclusion:**

The number of work breaks and hours spent at the computer seem to have a greater effect on NP than the place of work (office, at home), measurement time point (before COVID-19, during lockdown) or the workstation ergonomics. Further research should investigate the effect of social and psychological factors.

**Trial registration:**

ClinicalTrials.gov, NCT04169646. Registered 15 November 2019—Retrospectively registered, https://clinicaltrials.gov/ct2/show/NCT04169646.

## Background

The COVID-19 pandemic has suddenly forced around 50% of employees in Switzerland into a working from home setting during March and April 2020 [1]. In 2019, by comparison, only 24.6% of employees worked from home at least once a month and only a fraction (3%) worked predominantly from home [2]. Initial studies claimed that working from home during the COVID-19 pandemic was often performed at poorly designed workstations (49% out of 1100 respondents [3, 4]). Moreover, evidence indicates that regular break schedules were reduced such that office workers were taking fewer breaks during their work than before the lockdown (34% agreement [3]). In terms of workload, office workers experienced either under- or overwork, depending on their tasks and responsibilities [5, 6]. There are emerging reports that approximately one out of three office workers are more regularly performing overtime at home than previously in their offices before the lockdown [3, 4, 6].

COVID-19-related working from home appears to have changed the work experiences of office workers considerably. Positive changes (e.g., better work life balance, lower commuting demands) accompany the negative ones (e.g., loss of social contact with colleagues and supervisors, interruptions [1, 3, 6, 7]). Among the negative consequences related to working from home in times of the COVID-19 pandemic, an increase in non-specific neck pain (NP) has been reported [8, 9]. These findings need to be confirmed with higher levels of evidence, which is driver for this paper.

NP is a global burden of disease [10, 11]. In the workforce, especially among office workers, NP is epidemic [12]. Risk for NP and resources to reduce risk among office workers are multifactorial, including ergonomic, physical, psychological and psychosocial [13]. Among work-related risk factors, poor ergonomics (i.e., keyboard position close to the body, poor computer workstation design and work posture, sedentary work behaviours), high job stress, and low satisfaction with the workplace environment have been identified as risk factors in recent reviews and a longitudinal study [13–15]. Recently, some evidence for long working hours and prolonged sitting as risk factors for occupational NP has been reported [14, 16]. While long breaks during work do not seem to lower the risk of NP, evidence for frequent short breaks is weak to moderate [17, 18].

The aim of this study was to investigate the effect of the forced working from home situation during the COVID-19 pandemic on NP. We hypothesized that COVID-19-related working from home would increase NP as measured by NP intensity and neck disability. Secondly, we hypothesized that poor workstation ergonomics, the number of breaks at work, and long working hours at a computer would be associated with higher NP intensity and neck disability.

## Methods

### Design and participants

**Fig. 1:**
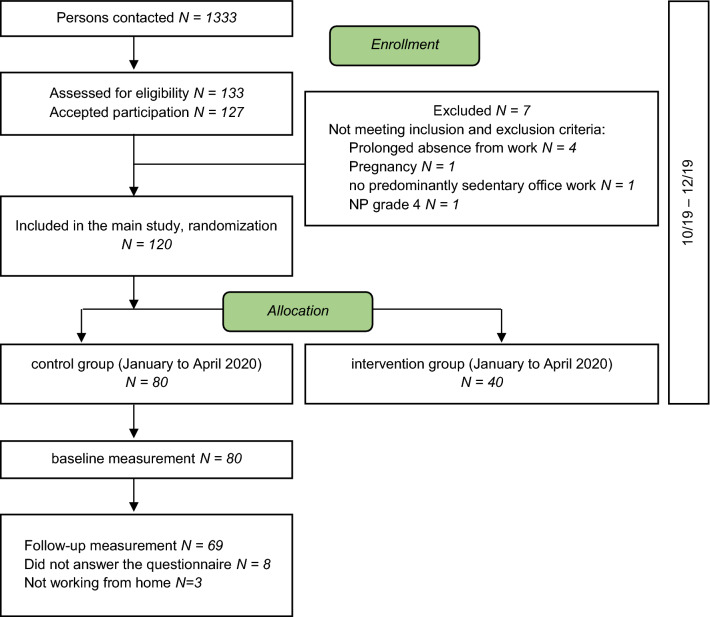
Flow-chart

This is a longitudinal study based on data from an ongoing stepped-wedge cluster randomized controlled trial (RCT) [19]. The study was approved by the Ethical Commission of the Canton of Zurich, Switzerland (Swissethics No. 2019-01678). Participants were recruited from two Swiss organisations in the Cantons of Zurich and Aargau between October and December 2019. Inclusion criteria were Swiss office workers aged 18–65 years, working more than 25 h per week (0.6 full-time equivalent) in predominantly sedentary office work, able to communicate in German (written, spoken), and provided written informed consent. Exclusion criteria were severe health conditions such as previous trauma or injuries of the neck, inflammatory disease, any history of cervical spine surgery or if exercise was contraindicated [19]. For this analysis, only those participants in the control cohort (control cluster, similar to a waiting list) between January and April 2020 (*n* = 80) who answered the COVID-19-related questions in full (*n* = 72 out of 80) and were working from home at the time of follow-up (*n* = 69 out of 72) were included (Fig. 1).

### Outcomes and measures

The association of working from home with NP was analysed. The primary outcome of NP was assessed with a measure of intensity (severity) and the level of disability. The mean intensity of NP over the last four weeks was rated on a numeric rating scale (NRS) scored from 0 (no pain) to 10 (maximum pain), and the neck disability, measured with the neck disability index (NDI) scored from 0 (no disability) to 100 (high disability). Both the NRS and NDI are validated assessment instruments for use in NP populations [20, 21]. Clinically relevant results were accepted with a minimal difference of 2.5 points on the NRS or 7 points in the NDI score [22, 23]. Secondary outcomes were number of breaks during work, time spent working at the computer (hours per day without lunch break), and self-rated quality of workstation ergonomics (overall rating, e.g. height of chair and table). Workstation ergonomics was rated by the study participants using a NRS scored from 1 (very good ergonomics) to 5 (very poor ergonomics). Furthermore, the participant characteristics, e.g. educational status, were collected.

### Procedure

Baseline data refers to work in the office, whereas follow-up data refers to working from home. Baseline data were collected with a 30-min online questionnaire administered in January 2020 ten weeks before the COVID-19 pandemic restrictions became effective in Switzerland, follow-up data in April 2020 during the fourth and fifth week of lockdown. On completion of the 30-min follow-up questionnaire of the main study in April 2020 (e.g., NDI, NP intensity, participant characteristics; see Aegerter et al. [19]), participants were invited to voluntarily answer another 20 COVID-19 related questions (5 to 10 min, e.g. working from home, workstation ergonomics at home and at the office). Information on workstation ergonomics in the office (i.e. at baseline before the pandemic) was collected retrospectively at follow-up. UNIPARK© (Berlin, Germany) was the platform used to host the online questionnaire.

### Statistical analysis

Participant characteristics at baseline and follow-up were analysed using descriptive statistics with mean values (including standard deviation), median, minimum, and maximum value, or in case of factor variables, relative and absolute frequencies. Wilcoxon Mann–Whitney rank sum tests were performed to investigate differences in participant characteristics between baseline and follow-up, as the assumption of normal distribution was not met.

Linear mixed effects models for repeated measures consider that measurements within a subject (participant) or time (baseline vs. follow-up) may be correlated. The strength of the evidence (confidence interval, p-value) might indicate that COVID-19-related working from home would increase or decrease NP as measured by NP intensity and neck disability. Two linear mixed effects models were fitted to the data to estimate the change in NP. The intensity of NP was the criterion variable in the first model, whereas NDI score was the criterion variable in the second model [Eqs. (1) and (2)]. The fixed effects were the same for both models: workstation ergonomics, working hours at the computer, number of breaks during work, and time. Time was included in the model to estimate whether the different measurement time points (baseline, follow-up) may have an effect on the criterion variables. An assumed normally distributed between-subject variation with zero mean and a variance for the subject’s σ^2^_ID_, with ID as subject identification, was included as a random effect. Furthermore, the model contained an assumed normally distributed within-subject variation with zero mean and a variance σ^2^ as an error term. Normality assumptions of between-subject and within-subject variation were verified graphically with quantile–quantile plots of random intercepts and residuals.

A sensitivity analysis extending the Eqs. (1) and (2) with the variables age and gender was performed for each linear mixed effect model. No interaction effects were integrated into the model. All analyses were performed in R using base and following analysis-specific packages: lme4 and multcomp [24]. Significance level alpha was set at 0.05. The p-values are expressed as the strength of evidence with little or no evidence (*p* > 0.1), weak evidence (0.05 < *p* ≤ 0.1), evidence (0.01 < *p* ≤ 0.05), strong evidence (0.001 < *p* ≤ 0.01) and very strong evidence (*p* ≤ 0.001) [25]. The data analyst was blinded to the identity of the participants.

The STROBE Statement checklist was used to guide the reporting of the study [26].

1 $${\text{NPintensity}}_{ij} = \, \beta_{0} + \, \beta_{1} \times {\text{ workstationergonomics}}_{ij} + \, \beta_{2} \times {\text{ computerworkhours}}_{ij} + \, \beta_{3} \times {\text{ workbreaks}}_{ij} + \, \beta_{4} \times {\text{ measurementtimepoint}}_{ij} + {\text{ ID}}_{i} + \, \varepsilon_{ij} ,{\text{with }}i \, = \, 1, \ldots , \, 69{\text{ subjects and }}j \, = \, 1 \, \left( {{\text{baseline}}} \right), \, 2 \, \left( {\text{follow - up}} \right)$$

2 $${\text{NDI}}_{ij} = \beta_{0} + \beta_{1} \times {\text{ workstationergonomics}}_{ij } + \beta_{2} \times {\text{ computerworkhours}}_{ij} + \beta_{3} \times {\text{ workbreaks}}_{ij} + \beta_{4} \times \, measurementtimepo{\text{int}}_{ij} + {\text{ ID}}_{i} + \, \varepsilon_{ij} ,{\text{with }}i \, = \, 1, \ldots , \, 69{\text{ subjects and }}j \, = \, 1 \, \left( {{\text{baseline}}} \right), \, 2 \, \left( {\text{follow - up}} \right)$$

## Results

Data from 69 participants were analysed with 11 excluded from the analysis due to absent responses on the COVID-19-related questions (*n* = 8) or not working from home (*n* = 3, Fig. 1). The descriptive statistics of the outcomes at baseline and follow-up are shown in Table 1. About three-quarters of participants were female (71.01%, *n* = 49). Seventy-eight percent of participants had tertiary level education (*n* = 54), 20.29% (*n* = 14) completed upper secondary education and 1.45% (n = 1) primary compulsory education. The mean age was 42.20 years at baseline (SD = 9.00 years). At baseline, the average BMI was 23.53 kg/m^2^ (SD = 3.47 kg/m^2^), whereas it was 23.71 kg/m^2^ at follow-up (SD = 3.42 kg/m^2^). The average of time between completion of the questionnaires was 101.30 days (SD = 7.91 days). There was no statistical evidence for a difference in the outcomes between baseline and follow-up (Table 1), except for workstation ergonomics *(p*-value < 0.0001, very strong evidence).

**Table 1:** Participant characteristics at baseline (work in the office) and follow-up (work at home)

	Baseline (*N* = 69)	Follow-up (*N* = 69)
*Neck disability index [NDI]*		
Mean (SD)	11.71 (10.14)	11.10 (10.80)
Median (Min, Max)	12.00 [0.00, 52.00]	12.00 (0.00, 52.00)
*Neck Pain (NP) intensity [NRS]*		
Mean (SD)	2.26 (1.86)	2.14 (2.19)
Median (Min, Max)	2.00 [0.00, 8.00]	2.00 [0.00, 7.00]
*Work breaks [number per day]*		
Mean (SD)	2.38 (0.91)	2.54 (0.96)
Median (Min, Max)	2.00 [1.00, 6.00]	3.00 [0.00, 5.00]
*Workstation ergonomics [NRS]*		
Mean (SD)	1.93 (0.77)	3.35 (0.98)
Median (Min, Max)	2.00 [1.00, 5.00]	3.00 [2.00, 5.00]
*Computer work [hours per day]*		
Mean (SD)	7.46 (1.29)	7.58 (1.19)
Median (Min, Max)	7.00 [5.00, 9.00]	7.00 [5.00, 9.00]

Neck disability index scored from 0 (no disability) to 100 (high disability); Neck Pain (NP) intensity scored with the numeric rating scale ranging from 0 (no pain) to 10 (maximum pain) and workstation ergonomics scored on a numeric rating scale ranging from 1 (very good ergonomics) to 5 (very poor ergonomics)

The results of the linear mixed effects models are presented in Tables 2 and 3. NP intensity was 0.68 points lower at follow-up compared to baseline (95% CI ranging from -1.35 to 0.00, evidence), indicating a slightly lower NP intensity during the lockdown. There was strong evidence that each working hour spent at the computer increased NP intensity by 0.36 points (95% CI ranging from 0.09 to 0.62), when all other covariates remained constant. In addition, for every point higher (i.e. worse) in the quality of workstation ergonomics, the intensity of NP increased by 0.35 points (95% CI ranging from 0.02 to 0.75, evidence). There is no evidence of an association of number of work breaks with NP intensity.

**Table 2:** NP intensity

	Coefficient	95% Confidence interval	*p* Value
Intercept	− 0.71	From − 2.93 to 1.50	0.53
Workstation ergonomics	0.39	From 0.02 to 0.75	0.04
Hours of computer work	0.36	From 0.09 to 0.62	0.01
Number of work breaks	− 0.18	From − 0.53 to 0.18	0.33
Measurement time point (follow-up)	− 0.68	From − 1.35 to 0.00	0.05

Estimated coefficients of workstation ergonomics, hours of computer work, number of work breaks, and measurement time point

**Table 3:** NDI

	Coefficient	95% Confidence interval	*p* Value
Intercept	16.52	From 4.96 to 28.35	0.01
Workstation ergonomics	− 0.15	From − 2.09 to 1.79	0.88
Hours of computer work	0.13	From − 1.26 to 1.51	0.86
Number of work breaks	− 2.30	From − 4.18 to − 0.42	0.02
Measurement time point (follow-up)	− 0.05	From − 3.68 to 3.59	0.98

Estimated coefficients of workstation ergonomics, hours of computer work, number of work breaks and measurement time point

Data presented in Table 3 shows that for each work break, the NDI score reduced by 2.30 points (95% CI ranging from  − 4.18 to  − 0.42, evidence), when all other covariates remained constant. There was no evidence of an association of workstation ergonomics, hours of computer work or measurement time point with neck disability.

All model assumptions were met. The log-likelihood test was not significant. A sensitivity analysis showed no evidence for an effect of gender or age on the results. There was no difference in the baseline data of the participants (*n* = 69) compared to those excluded (*n* = 11).

## Discussion

### Summary of findings

Our data yielded no evidence that neck disability, number of work breaks, or number of hours of computer work changed between pre COVID-19 pandemic (working at the office) and follow-up during the lockdown (working from home). However, we found evidence of a 0.68-point reduction in NP intensity during the lockdown. The number of hours working on a computer and the quality of workplace ergonomics may have an increasing effect on NP intensity, whereas the number of daily work breaks may decrease neck disability. There is strong evidence that workstation ergonomics was poorer when working from home compared to work in the office, but no association of time point of measurement with neck disability was found.

### Interpretation and comparison with literature

Overall, our findings are consistent with results previously presented in the literature [15–17]. In contrast to a recent report [9], our first hypothesis, that COVID-19-related working from home would increase NP intensity and neck disability, was not confirmed. Instead, NP intensity seemed to have decreased during the lockdown by slightly less than one point on the NRS. This could be due to the low level of NP intensity and neck disability at baseline. In our sample the prevalence of NP was very high (79%) due to the inclusion criteria, albeit low in severity (mean NRS 3.06 at baseline, NRS 2.81 at follow-up). It is more difficult to find a difference in people who are already mildly affected, as the measurement tool chosen (NRS) is not sufficiently sensitive to change [27]. Moreover, pain is multidimensional experience and is episodic, which means that not only disability and intensity (severity) but also frequency, duration, quality, localisation, and extent must be considered in NP analysis. Another possible reason for the findings could be the short follow-up time frame. Although there is evidence that a difference in NP can be observed after only a few weeks [28], the period of working from home might not have been enough long to cause a clear and clinically relevant change in NP. Therefore, it would be interesting to investigate potential long-term changes such as 12 months.

The second hypothesis, that poor workstation ergonomics, the number of breaks at work, and long working hours at a computer would be associated with higher NP intensity and neck disability was partially confirmed. Although Côté et al. [15] found no association of poor workstation ergonomics with NP intensity and neck disability, other studies have reported a negative association between the number of work breaks with NP intensity and neck disability [17], and a positive association for hours of computer work with NP intensity [16] which was confirmed in our analysis. Discrepancies in findings could be due to the method of assessing these outcomes (e.g., self-reported), the time frame and a small sample size.

Other findings of interest might be, that the number of breaks at work and the number of working hours did not increase significantly during the lockdown. This could be due to organisations as well as occupational health and safety regulators proactively managing the risk for injury by providing ideas and tips on how to stay well while working from home via various channels [29]. It is possible that the statistically significant worsening of workstation ergonomics during working from home may have been effectively counterbalanced by a decrease in risk factors or an increase of resources such as social support at work which was not measured in our study [30].

According to the biopsychosocial model of health, other factors such as biological (e.g., neck muscle endurance, physical activity level), psychological (e.g., job stress), or social (e.g., relationships) could have a greater effect on NP than the predictors analysed in this paper [31]. One possible assumption would be that most people may have experienced a decrease in work satisfaction and general well-being as well as a loss of communication and social exchange with colleagues and supervisors during the lockdown [7]. Compared to the work in an office, working from home requires greater self-regulation, work organisation (e.g., time structure), technical skills (e.g., new online tools), and a better distinction between work and private life (e.g., psychological detachment from work, work-family issues) [7, 29, 32]. As an example, it may happen that the employees notice too late that they are tired and need a work break. In addition, the duration and activity during the break might have changed while working at home, and the working time in general might have changed to be more flexible.

Another issue to consider is mental health and psychological distress, as there is evidence that people have become lonelier and more depressed while working from home [33, 34]. Compared to other samples, our study population is highly educated and was not challenged by an increase in job insecurity before, during, or after lockdown [29]. An important factor might be that the workers did not have to commute and so they had more leisure time (e.g., change in sleep duration or physical activity level, [35]). Hence, the current study may have underestimated the effects on NP during lockdown.

## Limitations

All office workers were employed by the local government. There were no reduced working hours during the lockdown and the level of employment (full time vs. part time) did not change. Therefore, the results cannot be generalised to office workers in the private sector, where the COVID-19 pandemic may have led to substantial changes in work organisation and increased unemployment and job insecurity.

In this analysis, social desirability bias cannot be excluded, as all data were collected using an online questionnaire (subjectivity). The measurement time point after five weeks in lockdown may have been insufficient to change NP (dose–response). The quality of workstation ergonomics at the office was assessed retrospectively at the time of follow-up, which may have led to recall bias. Moreover, no objective criteria for assessing workstation ergonomics were provided (e.g. correct height of the table), which may have led to biased results.

Non-responder bias was potentially small with a participation rate of 86% (*n* = 69 out of 80). There are three possible explanations for this rate. Firstly, only participants who completed the additional questions after the 30-min main questionnaire were included for analysis. This method was chosen to minimise the impact on the response rate of the ongoing RCT. Secondly, it is likely that intervention studies show higher drop-out rates during COVID-19, driven, among other things, by the sociodemographic and health status of the participants. Thirdly, as the ongoing RCT is a stepped-wedge design, all participants will receive the intervention. Thus, allocation to the control group is unlikely to have affected responses to the questionnaires or the response rate (responder-bias). In addition, the baseline levels of NP did not differ between participants and those excluded. Nevertheless, the sample size in this analysis is rather small. Sample size, corresponding statistical power, and the greater than expected dropout rate in our sample may have led to decreased power in detecting a true effect in our sample.

### Clinically relevance

Overall, the coefficients of the linear mixed effects models are very small. To achieve a clinically relevant change in NP intensity of at least 2.5 points, the change on the respective scale (covariate) must differ by several units; e.g. a reduction of computer work of seven hours. The effect of workstation ergonomics on NP intensity is not considered clinically relevant. A greater change would be necessary than is possible on the corresponding scale (seven points on a five-point scale). The reduction of 0.68 points on the NRS at follow-up is also not considered clinically relevant as it does not exceed the minimum detectable change of 1.5 points [36]. In contrast, three additional work breaks are required for a clinically relevant change in neck disability (7 points).

### Implications

In general, the number of work breaks and time spent at the computer seem to have a greater effect on NP than the place of work (at home vs. at the office), measurement time point (before the COVID-19 pandemic vs. during the lockdown), or the workstation ergonomics. It therefore seems important to inform and raise awareness on these two aspects, rather than about the acquisition of ergonomic equipment to improve NP.

### Further research

Further dimensions of pain, such as frequency, duration, location, quality, or extent would need to be investigated to enable more comprehensive statements about NP. The effect of psychosocial factors, such as mental health, or aspects such as commuting should be assessed in future studies. In other study populations, job insecurity could also play a significant role. Studies with a larger sample size or a longer follow-up phase are highly recommended, both of which will be difficult, as the collection of data prior to the COVID-19 pandemic will often result in high recall bias. With regard to work breaks, the duration of these breaks as well as their type (e.g. active vs. passive work breaks) should be investigated. Further studies should also consider variables of family structure especially in view of the closures of schools and day care centres introduced during the pandemic [29].

## Conclusion

COVID-19 pandemic forced many office workers to work from home. In this study, we investigated the effect of working from home on NP intensity and neck disability among office workers and found no evidence for a clinically relevant change in NP after five weeks of working from home. The place of work (at the office or at home), measurement time point (before COVID-19 vs. during the lockdown) and workstation ergonomics had no clinically relevant effect on NP, neither the intensity nor the level of disability. However, we found evidence that three additional breaks during work might reduce the degree of neck disability. NP intensity was found to be increased by the numbers of hours working on a computer, although a clinically relevant change requires large changes in work hours (at least seven). With regards to further research, the effect of psychological and social factors should be investigated in more detail, as COVID-19 has changed everyday life, not only at the workplace.

## Data Availability

The dataset analysed during the current study is available from the corresponding author on reasonable request.

## Abbreviations

COVID-19: Coronavirus disease 2019
NDI: Neck disability index
NP: Neck pain
NRS: Numeric rating scale
RCT: Randomized-controlled trial

## Code availability

The code is available from the corresponding author on reasonable request (software: R).
